# Comprehensive Bioinformatics Analysis Reveals Hub Genes and Inflammation State of Rheumatoid Arthritis

**DOI:** 10.1155/2020/6943103

**Published:** 2020-08-03

**Authors:** Conglin Ren, Mingshuang Li, Weibin Du, Jianlan Lü, Yang Zheng, Haipeng Xu, Renfu Quan

**Affiliations:** ^1^The Third Clinical Medical College of Zhejiang Chinese Medical University, Hangzhou, Zhejiang 310051, China; ^2^The First Affiliated Hospital of Zhejiang Chinese Medical University, Hangzhou, Zhejiang 310002, China; ^3^Department of Orthopedics, Xiaoshan Traditional Chinese Medicine Hospital, Hangzhou, Zhejiang 311200, China

## Abstract

Rheumatoid arthritis (RA) is an autoimmune disease characterized by erosive arthritis, which has not been thoroughly cured yet, and standardized treatment is helpful for alleviating clinical symptoms. Here, various bioinformatics analysis tools were comprehensively utilized, aiming to identify critical biomarkers and possible pathogenesis of RA. Three gene expression datasets profiled by microarray were obtained from GEO database. Dataset GSE55235 and GSE55457 were merged for subsequent analyses. We identified differentially expressed genes (DEGs) in RStudio with limma package, performing functional enrichment analysis based on GSEA software and clusterProfiler package. Next, protein-protein interaction (PPI) network was set up through STRING database and Cytoscape. Moreover, CIBERSORT website was used to assess the inflammatory state of RA. Finally, we validated the candidate hub genes with dataset GSE77298. As a result, we identified 106 DEGs (72 upregulated and 34 downregulated genes). Through GO, KEGG, and GSEA analysis, we found that DEGs were mainly involved in immune response and inflammatory signaling pathway. With the help of Cytoscape software and MCODE plug-in, the most prominent subnetwork was screened out, containing 14 genes and 45 edges. For ROC curve analysis, eight genes with AUC >0.80 were considered as hub genes of RA. In conclusion, compared with healthy controls, the DEGs and their closely related biological functions were analyzed, and we held that chemokines and immune cells infiltration promote the progression of rheumatoid arthritis. Targeting the eight biomarkers we identified may be useful for the diagnosis and treatment of rheumatoid arthritis.

## 1. Introduction

Rheumatoid arthritis (RA) is an autoimmune disease, mainly destroying synovium and joints, characterized by autoantibodies that target immunoglobulin G (known as rheumatoid factor, RF) and citrullinated proteins (called anticitrullinated protein antibodies, ACPAs) [1]. Some epidemiological studies conducted in western countries showed that the prevalence of rheumatoid arthritis is about 0.5-1.0% [2, 3]. Rheumatoid arthritis is a complicated disease due to the changeable clinical manifestations and complications in different patients or disease stages, which brings difficulties to the clinical work of doctors.

The serological detection of autoantibodies is a crucial indicator in the diagnosis and prognosis of rheumatoid arthritis, but about 25% of patients are seronegative and thus may experience a delay in diagnosis as well as initiation of drug therapy [4]. Moreover, it was estimated that 50% of seropositive patients had negative serum test results at the beginning of the disease [5]. Previous studies have shown that proinflammatory cytokines in inflammatory synovium, such as interleukin-8, can stimulate osteoclasts proliferation and then result in bone resorption of RA patients [6–8]. However, some scholars found that bone destruction may also occur in ACPA-positive individuals without detectable inflammation conditions [9]. A recent study supporting the latter result demonstrated that monoclonal ACPAs derived from B cells in the synovial fluid of RA patients have obvious epitope specificity, which promotes the differentiation of osteoclasts in cell cultures [10]. Although the pathogenetic insights, classification criteria, and therapeutic strategies of RA have been updated in the past 20 years, some patients are still unable to achieve satisfactory clinical remission or have serious adverse reactions to antirheumatoid therapy, so more efforts are required to address these unmet needs.

The microarray technology has emerged for more than 20 years, which makes it possible to analyze the complete transcriptional information of various cell types and tissues [11]. Studies based on gene expression analysis have obtained new findings, elucidating how the transcriptome varies among distinct phenotypes and stages of disease [12, 13]. The Gene Expression Omnibus (GEO) is a user-friendly repository, in which stores microarray, next-generation sequencing, and other forms of genomics data for users to query and download. Here, we aimed to dissect biomarkers and inflammation state of rheumatoid arthritis by comprehensively applying multiple bioinformatics analysis tools including R packages from Bioconductor, STRING database, CIBERSORT website, Cytoscape, and GSEA software. The findings in our study may contribute to novel ideas for better diagnosis and treatment of rheumatoid arthritis.

## 2. Materials and Methods

### 2.1. Data Download and Processing

Three microarray datasets GSE55235, GSE55457 [14], and GSE77298 [15] were obtained from the GEO database (https://www.ncbi.nlm.nih.gov/geo). A total of 20 normal synovial tissues and 23 diseased specimens were enrolled from dataset GSE55235 and GSE55457, whose detection platforms were identical (GPL96, HG-U133A). The dataset GSE77298 was based on GPL570 platform (HG-U133 Plus 2), containing 7 synovium samples from healthy controls (HC) and 16 from RA patients. According to the research plan, the former two datasets were merged as training dataset to explore hub genes, and mRNA profiles of GSE77298 were used to assess whether the discovered hub genes have excellent diagnostic value for RA.

Data processing was divided into four steps. First, the three probe expression matrix files (∗series_matrix.txt) downloaded from GEO database were normalized and log2 transformed. Next, we matched the platform annotation file with each probe expression matrix and well-annotated probes were retained. For multiple probes corresponding to one gene, the average expression value was taken for further analysis. Third, we merged the expression matrix of GSE55235 and GSE55457 into one, and the order of samples in dataset was rearranged. Last, R package sva, installed from Bioconductor (https://bioconductor.org/), was applied to eliminate the heterogeneity caused by different experimental batches and platforms.

### 2.2. Identification of DEGs

Differentially expressed genes (DEGs) were screened out by limma package [16] based on the comparison of expression values between HC samples and RA samples. The screening criteria for DEGs were as follows: log2 fold change (FC) should be greater than 2 or less than -2 and adjusted *p* value <0.05. The analysis results were presented by heatmap and volcano map drawn in RStudio software (version:1.2.1335).

### 2.3. Functional Enrichment Analysis

The Bioconductor package clusterProfiler [17] was applied to carry out Gene Ontology (GO) and KEGG pathway analysis for DEGs. Based on the threshold *p* value <0.05, GO terms and signal pathways with significant enrichment were screened out. Gene Set Enrichment Analysis (GSEA) is a software codeveloped by UC San Diego and Broad Institute [18, 19], which assesses whether a predefined gene set shows statistically significant differences between two biological phenotypes (e.g., HC and RA). Expression dataset collapsed to gene symbol and phenotype information were uploaded to GSEA for enrichment analysis with default parameters. Hallmark gene sets (h.all.v7.1.symbols.gmt) selected in the present study were downloaded from Molecular Signatures Database (MSigDB) [20, 21]. Enrichment results with nominal *p* value <0.01 as well as FDR <0.25 were considered statistically significant.

### 2.4. Immune Infiltration Analysis

Online analysis tool CIBERSORT(https://cibersort.stanford.edu/), a method for calculating the cell proportion of complex tissues based on gene expression profiles, is superior to other methods in terms of large-scale analysis of RNA mixtures [22]. In this study, we used CIBERSORT to characterize the inflammation state of RA and healthy joint tissue with default signature gene file (22 types of immune cells). The analysis result was filtered according to *p* value <0.05, and the immune cell composition of each sample was shown in barplot.

### 2.5. PPI Network Construction and Module Analysis

In order to explore the mutual relationship between proteins encoded by different genes, DEGs were imported into STRING website (version:11.0) for further analysis [23]. The lowest interaction score should be greater than 0.4 and isolated nodes in the network were removed. Next, we output the analysis results to a TSV format file and used Cytoscape software (version:3.7.1) for details processing and module analysis. MCODE [24] is a plug-in downloaded from Cytoscape App Store, which can find closely connected nodes in a complex network based on topology. Therefore, we applied this plug-in to detect critical modules in PPI network with default parameters.

### 2.6. Verification of Hub Genes by ROC Analysis

Genes in the most significant module identified by MCODE plug-in were selected as candidate hub genes. To evaluate the role of candidate genes in the diagnosis of RA, receiver operating characteristic (ROC) curve analysis was conducted in RStudio with pROC package [25]. The genes with area under curve (AUC) >0.8 as well as *p* value <0.05 were considered as hub genes of RA.

## 3. Results

### 3.1. Identification of DEGs

**Figure 1:**
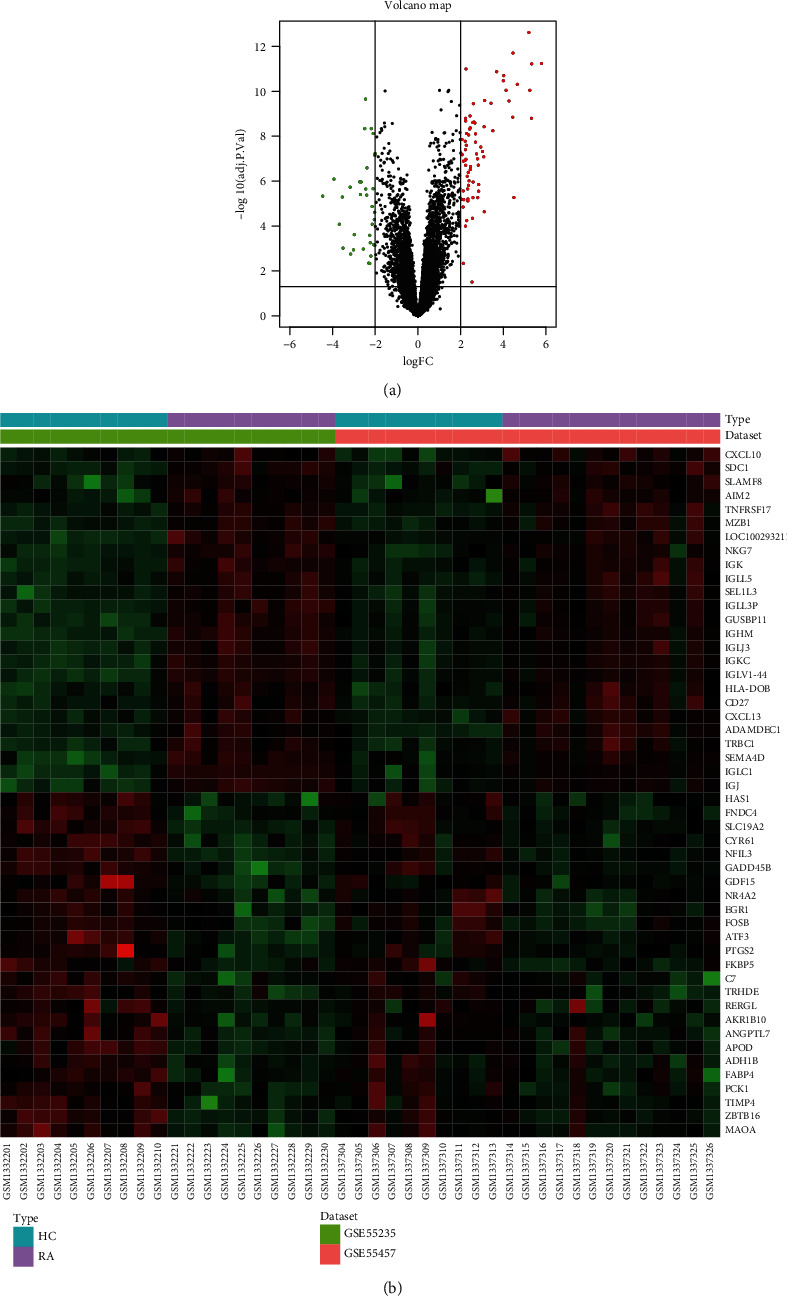
Visualization of differentially expressed genes (DEGs). (a) DEGs screened by threshold (adjusted p value <0.05 and |logFC| >2) were presented by volcano map. (b) Heatmap showed the expression of top 25 upregulated and downregulated genes ordered by adjusted *p*-value.

By using limma package to analyze the differential expression of the integrated dataset, we obtained 106 DEGs composed of 72 upregulated and 34 downregulated genes. DEGs screened by threshold were visualized by volcano map (Figure 1(a)). The expression of top 25 upregulated and downregulated genes ordered by adjusted *p* value in each sample was shown by heatmap (Figure 1(b)).

### 3.2. GO and KEGG Enrichment Analysis

**Figure 2:**
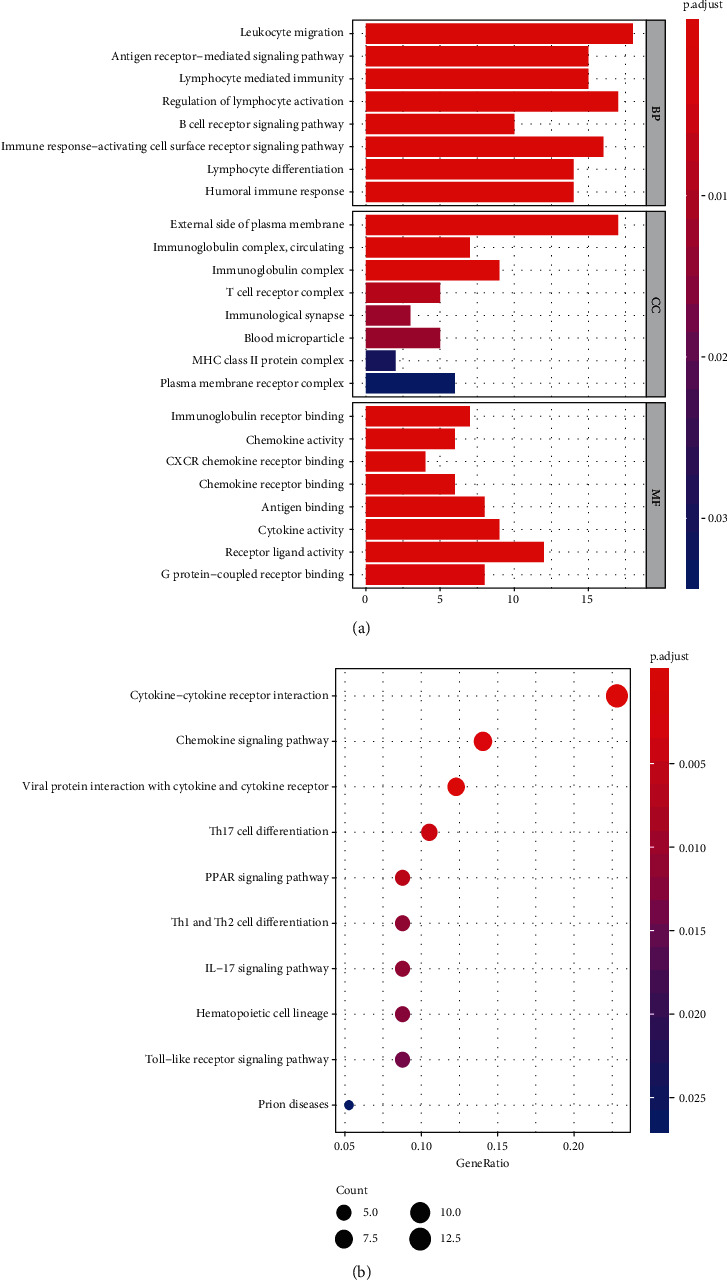
Results of functional enrichment analysis. (a) GO analysis results of DEGs, top 8 terms of each category were listed. (b) The top 10 pathways of KEGG analysis.

Enrichment analysis conducted by clusterProfiler package revealed the biological functions and pathways related to DEGs. As is shown in Figure 2(a) and Table 1, GO annotation of DEGs consists three parts (BP, CC, MF) and top 8 terms of each category were listed. GO analysis results revealed that the biological functions of DEGs were mainly related to immune cells infiltration and inflammatory signaling pathway. The top 10 pathways of KEGG analysis (Figure 2(b) and Table 2) indicated that DEGs were involved in signal transduction and inflammatory response-related pathways, such as chemokine signaling pathway, cytokine-cytokine receptor interaction, Th cells differentiation, IL−17 signaling pathway, and Toll-like receptor signaling pathway.

### 3.3. GSEA Analysis

**Figure 3:**
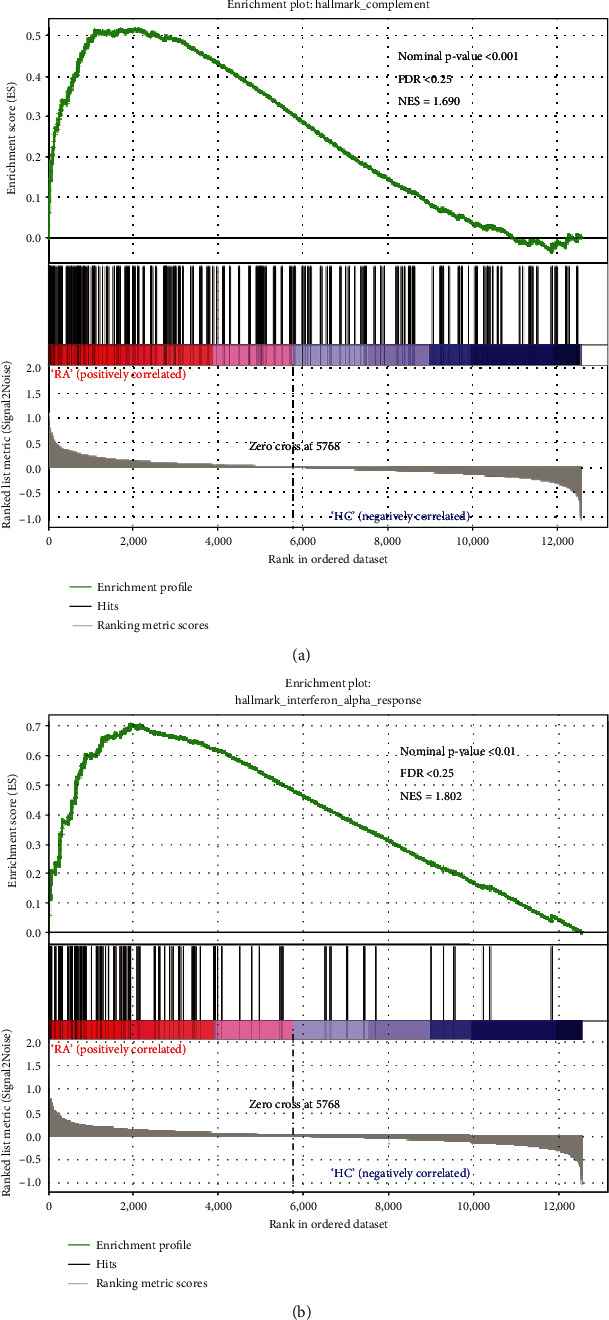
GSEA analysis of DEGs. (a) Enrichment plot for complement. (b) Enrichment plot for interferon alpha response.

The overall expression data and hallmark gene sets were loaded into GSEA software for further analysis. Hallmark gene sets contain 50 gene sets, representing well-annotated biological functions or processes determined by integrating multiple MSigDB datasets. According to the filter threshold of analysis results, complement and interferon alpha response gene sets were significantly upregulated in RA samples. Normalized Enrichment Score (NES) and nominal *p* value were presented in the upper right corner of the plot (Figures 3(a) and 3(b)).

### 3.4. Immune Infiltration Analysis

**Figure 4:**
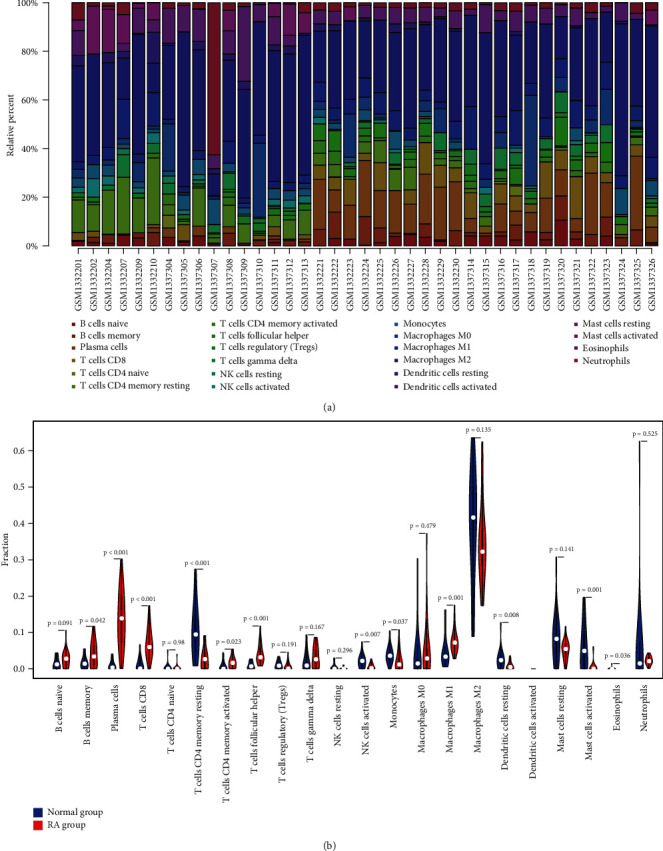
Immune infiltration analysis performed by CIBERSORT. (a) Barplot showed the composition of immune cells in 23 RA samples and 16 normal samples. (b) The content of 22 types of immune cells in HC (blue color) and RA (red color) samples was compared. *p* value <0.05 was considered statistically significant.

CIBERSORT analytical tool can accurately calculate levels of 22 types of leukocyte subpopulations in synovial tissues profiled by microarray. Based on significance threshold *p* value <0.05, 4 samples (GSM1332203, GSM1332205, GSM1332206, GSM1332208) were abandoned.  Figure 4(a) shows the composition of immune cells in 23 RA samples and 16 normal samples. Wilcoxon test was conducted to determine whether there was a significant difference in immune cell infiltration between RA and HC samples. In violin plot (Figure 4(b)), it was revealed that memory B cells, plasma cells, CD8 T cells, activated CD4 memory T cells, T cells follicular helper, monocytes, and macrophages M1 were abundant in RA synovial membrane.

### 3.5. PPI Network Construction and MCODE Plug-in Analysis

**Figure 5:**
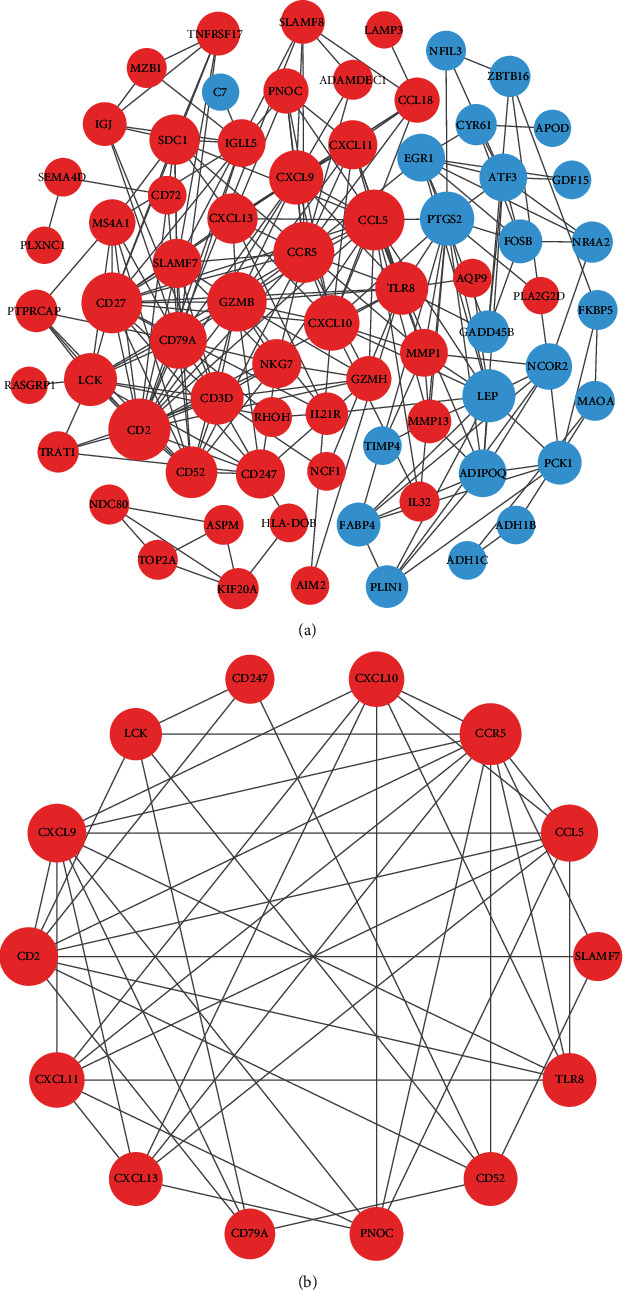
PPI network construction and module analysis. (a) The PPI network of DEGs was constructed in Cytoscape. (b) The most significant module was obtained by MCODE plug-in. Upregulated genes were marked with red color, and downregulated genes were blue. The diameters of nodes were positively correlated with their connectivity degree.

PPI network constructed by STRING database was adjusted and visualized by Cytoscape (Figure 5(a)). Upregulated genes were marked with red color, and downregulated genes were blue. The diameters of nodes were positively correlated with their connectivity degree. In order to figure out the core modules of complex network, we performed MCODE plug-in analysis and identified 5 modules. The most significant module with the highest score (module score:6.923) was shown in Figure 5(b), containing 14 genes and 45 edges.

### 3.6. Validation of Hub Genes with GEO Database

**Figure 6:**
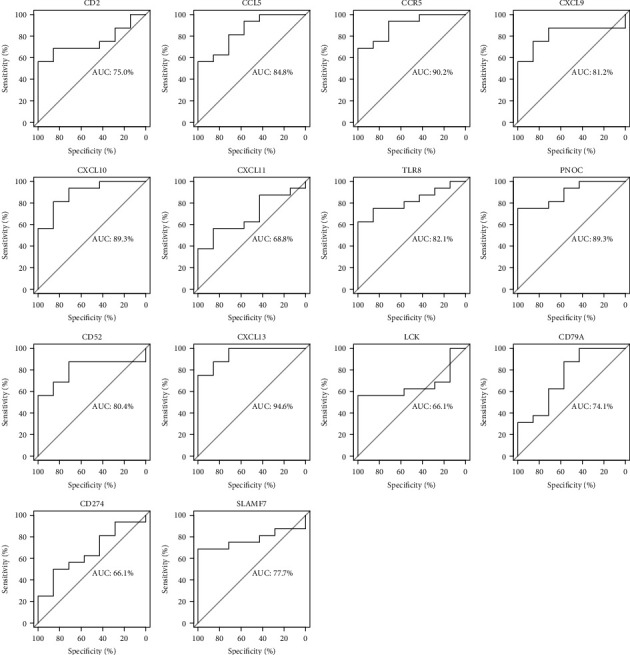
Validation of candidate hub genes by ROC curve analysis. Among the 14 genes screened out by MCODE plug-in, eight genes with AUC more than 0.80 were considered as hub genes of RA.

The 14 genes screened by MCODE plug-in, which could be used to distinguish RA patients from healthy controls, were considered as candidate hub genes. To further validate the expression of these 14 genes in synovium of other patients, we selected GSE77298 as testing dataset and performed ROC analysis in RStudio. The analysis results were available in Figure 6. Among the 14 genes, eight genes (CCR5, CCL5, CXCL9, CXCL10, CXCL13, PNOC, TLR8, and CD52) with AUC more than 0.80 were considered as hub genes, indicating that they have the capability to diagnose RA patients with excellent specificity and sensitivity.

## 4. Discussion

Rheumatoid arthritis is an autoimmune nature joint disease with irreversible cartilage destruction and bone erosion [1]. If not treated promptly and effectively, RA can seriously reduce the quality of life and even cause disability. There is no doubt that understanding diseases at the molecular level will help to improve their diagnosis and treatment [26, 27]. Up to now, various biomarkers have been identified to be associated with rheumatoid arthritis and may be selected as therapeutic targets, but the detailed mechanism of gene regulation leading to disease progression remains elusive [28, 29].

In our study, we aimed to identify biomarkers of RA and uncover their biological functions through bioinformatics analysis. Dataset GSE55235 and GSE55457 were selected as training dataset in our analysis. As a result, 72 upregulated and 34 downregulated DEGs, at least 4-fold change between RA and normal samples, were screened out. Next, the DEGs were annotated by performing functional enrichment analysis, and we observed that these genes were closely related to immune response and inflammatory signaling, such as humoral immune response, chemokine signaling pathway, antigen receptor-mediated signaling pathway, Th17 cell differentiation, and IL-17 signaling pathway. Previous studies have shown that the infiltration of Th17 cells in synovium is a typical pathological change of RA [30, 31], which releases abundant IL-17A, IL-17F, IL-6, and mediates the activation of neutrophils. An animal experiment indicated that blockade of IL-17 can delay the destruction of articular cartilage by inhibiting local inflammatory reaction in the collagen-induced arthritis (CIA) mice [32]. In addition to cytokines, chemokines were also suggested to be involved in systemic inflammatory disorders [33–35]. It has been reported that elevated plasma chemokine level was discovered in RA patients, and CXCL10 can be served as a diagnostic biomarker for active rheumatoid arthritis [36]. Similarly, the GSEA results based on all gene expression information revealed that 2 gene sets (interferon alpha response and complement) were significantly enriched in RA phenotype at nominal *p* value <0.01. Considering the crucial role of inflammatory response and leukocyte infiltration in the pathogenesis of RA, we carried out CIBERSORT analysis to explore the differences between two groups in the distribution of immune cells. Compared with healthy controls, memory B cells, plasma cells, activated CD4 memory T cells, T cells follicular helper, monocytes, and M1 macrophage were abundant in RA synovial membrane, which was consistent with the published studies [37, 38].

A PPI network of DEGs was established using STRING website and Cytoscape software. With the help of MCODE plug-in, we screened out the most significant subnetwork, which consists of 14 nodes and 45 edges. Moreover, we validated the above 14 genes by performing ROC analysis with the testing dataset GSE77298. As a result, 8 genes with *p* value <0.05 as well as AUC >0.80 showed excellent diagnostic value for rheumatoid arthritis, and thus were considered as hub genes of RA, including CCR5, CCL5, CXCL9, CXCL10, CXCL13, PNOC, TLR8, and CD52.

The chemokine system is a large and complicated family, containing more than 50 ligands and 25 receptors [39]. Chemokines can be divided into four subfamilies, known as CXC, CC, CX3C, and XC, according to the position difference of cysteine residues. Correspondingly, there are four types of receptors that can bind to their ligands and have the ability to trigger a G*α*i-mediated signaling pathway [40, 41]. In inflammatory diseases like rheumatoid arthritis, chemokines mediate the migration of leukocytes into synovial membrane, participating in angiogenesis, endothelial activation, synovial hyperplasia, and regulation of cartilage metabolism [42–45]. Pandya [36] found that chemokine CXCL9, CXCL10, CXCL13, CCL4, and CCL22 were significantly higher in the blood plasma of RA patients compared to healthy people by multivariate discriminant analysis. Besides, various studies based on targeting of chemokines and their receptors have been widely taken out for therapeutic purposes. Tofacitinib is a Janus kinase inhibitor and has been proved to inhibit chemokines secretion in synovium, including CXCL10, CXCL13, and CCL2 [46]. In animal experiments, the use of receptors inhibitors also achieved fine therapeutic effect. J­113863, an antagonist of CCR1, has positive effects on the CIA model of murine [47], and adjuvant-induced arthritis in rats can be inhibited by Met-RANTES, a drug blocking CCR1 and CCR5 [48]. All these findings suggested that chemokines act as a key factor in the pathological process of RA and deserve more attention.

TLR8, a gene encoding Toll-like receptor 8, was predominantly expressed in peripheral blood leukocytes, playing a fundamental role in antimicrobial immune responses and autoimmune inflammation [49]. TLR7 and TLR8 are located at the membranes of endosomal compartment, involved in recognizing viral RNA [50, 51]. Toll-like receptor signaling pathway, stimulated by the adaptor protein MyD88, modulates NFкB, IRF-7, and MAPK activation, resulting in the release of proinflammatory cytokines and cell adhesion molecules [52]. CD52, also known as EDDM5, is a low molecular weight glycoprotein that is abundant in B and T cells [53]. Alemtuzumab, the first therapeutic anti-CD52 antibody, has been shown to be effective in the treatment of autoimmune diseases such as RA and inflammatory bowel disease. However, in recent studies, alemtuzumab has been found to result in long-term immunosuppression, particularly depleting CD4+ T cells, and increase the risk of opportunistic infection [54, 55]. Therefore, in the future clinical practice, it is essential to pay attention to the dynamics of immune reconstitution and the results of immunosuppression. Unlike the genes discussed above, PNOC is rarely mentioned in previous rheumatoid arthritis studies, and thus needs more exploration. This gene encodes a preproprotein, which is the precursor of nociceptin. A previous study demonstrated that the mRNA expression of PNOC and NOP was suppressed by lipopolysaccharide as well as inflammatory cytokines [56]. In the current study, PNOC gene is upregulated in RA patients, which may stimulate the secretion of cytokines, further aggravate the inflammatory response of synovium and become a vicious circle.

## 5. Conclusions

In summary, we integrated multiple bioinformatics tools and found that chemokines and immune cell infiltration were extremely critical factors in the progression of rheumatoid arthritis. The eight hub genes we identified may serve as potential therapeutic targets for RA and further investigations are required to support our conclusions.

## Figures and Tables

**Table 1:** GO analysis results of DEGs (top 8 terms of each category were listed).

Ontology	ID	Description	Adj. *p* value	Count
BP	GO:0050900	Leukocyte migration	2.33E-08	18
BP	GO:0050851	Antigen receptor-mediated signaling pathway	2.33E-08	15
BP	GO:0002449	Lymphocyte mediated immunity	6.91E-08	15
BP	GO:0051249	Regulation of lymphocyte activation	6.91E-08	17
BP	GO:0050853	B cell receptor signaling pathway	2.54E-07	10
BP	GO:0002429	Immune response-activating cell surface receptor signaling pathway	3.05E-07	16
BP	GO:0030098	Lymphocyte differentiation	4.36E-07	14
BP	GO:0006959	Humoral immune response	4.36E-07	14
CC	GO:0009897	External side of plasma membrane	1.28E-08	17
CC	GO:0042571	Immunoglobulin complex, circulating	7.02E-06	7
CC	GO:0019814	Immunoglobulin complex	8.86E-06	9
CC	GO:0042101	T cell receptor complex	0.008265	5
CC	GO:0001772	Immunological synapse	0.012398	3
CC	GO:0072562	Blood microparticle	0.012678	5
CC	GO:0042613	MHC class II protein complex	0.030215	2
CC	GO:0098802	Plasma membrane receptor complex	0.033455	6
MF	GO:0034987	Immunoglobulin receptor binding	8.86E-06	7
MF	GO:0008009	Chemokine activity	1.34E-05	6
MF	GO:0045236	CXCR chemokine receptor binding	1.51E-05	4
MF	GO:0042379	Chemokine receptor binding	5.51E-05	6
MF	GO:0003823	Antigen binding	7.28E-05	8
MF	GO:0005125	Cytokine activity	7.80E-05	9
MF	GO:0048018	Receptor ligand activity	0.000201	12
MF	GO:0001664	G protein-coupled receptor binding	0.002229	8

**Table 2:** Top 10 pathways of KEGG analysis.

ID	Description	Adj. *p* value	Count
hsa04060	Cytokine-cytokine receptor interaction	1.44E-05	13
hsa04061	Viral protein interaction with cytokine and cytokine receptor	0.000459	7
hsa04062	Chemokine signaling pathway	0.002564	8
hsa04659	Th17 cell differentiation	0.003911	6
hsa03320	PPAR signaling pathway	0.005839	5
hsa04658	Th1 and Th2 cell differentiation	0.011245	5
hsa04657	IL-17 signaling pathway	0.011245	5
hsa04640	Hematopoietic cell lineage	0.01248	5
hsa04620	Toll-like receptor signaling pathway	0.013885	5
hsa05020	Prion diseases	0.026379	3

## Data Availability

The data that support the findings of this study are openly available.
